# Detecting changes in health and daily activities using environmental and electricity sensors and machine learning

**DOI:** 10.1007/s12553-026-01082-x

**Published:** 2026-05-26

**Authors:** Tamaryn Menneer, Tim Walker, Karen Spooner, Emma Bland, Lucia Pratto, Ian Wellaway, Mark England, Richard A. Sharpe, Catherine Leyshon, Markus Mueller

**Affiliations:** 1 European Centre for Environment and Human Health, University of Exeter Medical School, University of Exeter, Penryn Campus, Penryn, UK; 2 Environment and Sustainability Institute, University of Exeter, Penryn Campus, Penryn, UK; 3 Coastline Housing Ltd, Barncoose Gateway Park, Redruth, UK; 4 Research IT, University of Exeter, Streatham Campus, Exeter, UK; 5 Public Health, Cornwall Council, County Hall, Truro, Cornwall UK; 6 Centre for Geography and Environmental Science, University of Exeter, Penryn Campus, Penryn, UK; 7 Centre for Environmental Mathematics, Department of Earth and Environmental Science, Faculty of Environment, Science and Economy, University of Exeter, Penryn Campus, Penryn, UK

**Keywords:** Independent living, Health, Time series analysis, Machine learning, Indoor environment, Electricity usage

## Abstract

**Purpose:**

The purpose of this feasibility study was to test whether changes in the indoor environment and electricity usage can be used to detect changes in occupant activities that reflect changes in health.

**Methods:**

Temperature, relative humidity, carbon dioxide and electrical current were measured over 14+ months in 36 single-occupant homes. Data regarding health and household activities were collected via frequent optional surveys. Characteristics were extracted from the sensor data, including descriptive statistics, timings of peaks and troughs, frequency, deviations from average and modelled values (long short-term memory neural network). Machine learning was used to map these characteristics to occupant responses about health and activities, using a multilayer regressor neural network. Models were trained using all participant data together and separately on data from individual participants.

**Results:**

Detection of an existing health condition being worse than normal gave a balanced accuracy of 62%, with 95% of the models having performance above chance. For individual participants, highest performance for detecting worse health was 75% balanced accuracy. Models trained on this participant’s data generally gave the highest performance detecting specific reasons for worse health, with 80% for an existing health condition, 87% for mental health, and 84% for physical pain.

**Conclusions:**

Overall, the results from this feasibility study provide evidence that data from environmental and electrical-power sensors could be useful in monitoring health and supporting independent living at home. When indicators of worsening health are detected, interventions could be made, providing external support from service providers, or prompting behavioural change, in advance of the issue worsening.

**Supplementary Information:**

The online version contains supplementary material available at 10.1007/s12553-026-01082-x.

## Introduction

Supporting vulnerable or older adults who live independently at home allows health, wellbeing and autonomy to be maintained, and could therefore also reduce burdens on health and care services associated with urgent treatment or hospital visits in the UK [1], particularly given aging populations [2]. Developments in sensor technology can be beneficial in healthcare [3], including supporting independent living by using sensors to monitor vital signs [4, 5] or the home environment [6], with expected benefits for preventative care [7, 8]. Much research focusses on monitoring activities of daily living (ADLs), such as bathing and eating, to check for changes or the absence of certain activities. ADL detection is usually achieved with wearable devices, binary sensors (e.g., kettle on/off), pressure pads, motion sensors, or thermal imaging [9–15]. Such monitoring can decrease time spent in hospital [16]. However, these systems are often bespoke targeted approaches for a specific situation or health condition, and may require specialist installation or occupant interaction with the device (e.g., [15, 17]).

Instead, more widely available and less intrusive sensors could be used. Sensors for temperature, relative humidity (RH), carbon dioxide (CO_2_), and electricity usage are increasingly available and routinely installed by housing providers to monitor for unhealthy home environments (e.g., mould) [18, 19]. Using these existing sensors to monitor ADLs avoids installation of potentially costly specialist equipment and has minimal intrusion for the occupant [20].

However, the use of indoor environmental data for monitoring ADLs is an under-researched area. Temperature and humidity data are generally combined with other sensors, such as motion, location or sound sensors [21–24]. More broadly, CO_2_ can be used as a proxy for occupancy [25, 26], and is therefore applicable to inferring ADLs.

Conversely, there is much research on electricity usage data revealing changes in household activities [17, 27–31]. Monitoring specific appliances can reveal ADLs [32], with some approaches using non-intrusive electrical load monitoring to disaggregate appliance use associated with certain activities [20, 33–37], and some considering integration of appliance use into a health status dashboard [38]. Other methods use overall household electricity usage and characteristics such as times of maximum and minimum usage [29] or variance [39], to detect deviations from normal patterns.

Specific to health monitoring, electricity smart meter data has been used to infer ADLs to assess changes associated with dementia in a clinical trial with two patients [40], and to monitor decreases in frailty risk after interventions with 19 elderly participants [41].

Methods developed for monitoring ADLs in electricity consumption data include anomaly detection (e.g., [17, 28]). Time series analysis methods characterise features in the sensor data, such as descriptive statistics (e.g., [29]), frequency analysis [35], or difference from expected readings. Expected readings can be modelled using machine learning methods such as long short-term memory neural networks (LSTM) [28]. Autoregressive integrated moving average (ARIMA) models can also be used, but are often outperformed [39, 42, 43].

Having determined these sensor-data characteristics, machine learning can be used to map from the characteristics to anomalies. Methods include neural networks, support vector machines and cluster analysis, with outputs being a binary classification of anomaly versus normal or a magnitude of deviation from the normal using a regression approach ([44] provides a review).

This previous work has provided important fundamental proof-of-concept in using electricity data to monitor ADLs and support independent living. As such, these and similar studies are typically small-scale, or an intensive investigation into specific case data, with few studies directly applied to health, and some tested only on simulated events, or requiring specialised appliance monitoring. Therefore, an ongoing need remains to assess ADL monitoring in real-life settings for larger sample sizes over a long period of time [9].

The aim of this feasibility study was to test a novel approach for detecting changes in occupant health, with more participants and for a longer time than previous studies and using real responses about health. We use indoor environmental and electrical power consumption sensors to test whether these data can be used to detect changes in occupant activities that reflect changes in health. The current study purposively used off-the-shelf sensors that are easy to install, non-intrusive, relatively inexpensive, and may already exist in many homes. We employed the above time-series analysis approaches to characterise sensor data, and regressor neural networks to learn to identify changes in health.

## Terminology and Abbreviations

Table 1 provides terms and abbreviations used throughout.

**Table 1:** Terminology and abbreviations

Abbreviation	Definition
ADL	Activity of daily living
BA	Balanced accuracy, calculated as mean of true-positive and true-negative rates. Reported on a scale of 0 to 1 in the results tables, and as a percentage (0-100%) in the main text.
CO_2_	Carbon dioxide
IQR	Inter-quartile range
LSTM	Long short-term memory neural network
P_AC_	Proportion of 20 runs with performance ≥ 1.96 SDs above the chance-level mean value.
RH	Relative humidity
RMSE	Root mean square error
run	One implementation of a given neural network, with the same architecture and dataset, but a different split between training and test data.
sample	One row of data comprising one input and output pair for the neural network.
SD	Standard deviation
sensor-measure	The measure from the sensor: temperature, relative humidity, carbon dioxide, electrical power.
SMOTE	Synthetic minority oversampling technique

## Methods

### Participants

We recruited 37 participants during May 2023 to September 2023. Participants lived on their own and reported having a health condition that interferes with their daily routine. One participant withdrew for health reasons. Thirty-six participants completed the study with participation for at least 14 months. Nineteen were female and 17 male, with age range 53–87 years (mean = 71). All lived in social housing in Cornwall, UK, a typically under-served community [45–47].

During the first visit to participants’ homes, participants gave informed consent, questionnaires were administered, and sensors were installed.

### Health and activities data

Throughout the project, 32 participants received a text message (Short Message Service) or email approximately every week with a link to a brief optional survey. The remaining four participants declined receiving the survey. The survey link was sent 63 times. Participants could respond as to whether their health was worse, normal or better during the past week, then select reasons and days that health was worse. Participants could also respond about unusual events in their home during the past week, then select reasons and days.

The survey was answered from 0 to 70 times for a given participant, with 10 participants answering at least 50 times.

The selected responses were assigned a 1. Non-selected responses were assigned a 0 if the mutually exclusive response was selected. For example, ‘health worse’ and ‘health better’ responses were assigned 0 when the ‘health normal’ response was selected. There were nine survey question categories in total: ‘health worse’, ‘health better’, ‘existing health condition worse’, ‘physical pain worse’, ‘mental health worse’, ‘an unusual event has happened’, ‘less time at home than usual’, ‘more time at home than usual’, ‘maintenance or building work’, ‘a visitor to the home’.

A face-to-face questionnaire was administered when sensors were installed including questions on demographics, health and household daily routine. Questionnaires on health and routine were repeated over the phone at the middle and end of the participant’s involvement in the project. In the current study we only used responses about the time that the participant typically got out of bed.

Supplementary Materials Sect. 1 provides more detail.

### Sensors

Sensor systems were installed in each participant’s home for 14 to 18.5 months (mean = 16.8). Sensors measured air temperature, RH, CO_2_ and electrical power, with readings taken every 12–60 s, depending on the sensor. Readings were transmitted to the cloud, via a gateway that was typically placed in the living room or hall. Supplementary Materials Sect. 2 provides details.

Wherever possible, environmental sensors were placed in the living room, on furniture or on an internal wall, and away from draft or heat sources. The temperature and RH sensor was typically placed 1.5–2 m above the floor level, avoiding locations that receive direct sunlight. The CO_2_ sensor required a mains power socket, and was typically placed 30–90 cm above floor level.

### Sensor data processing

**Fig. 1:**
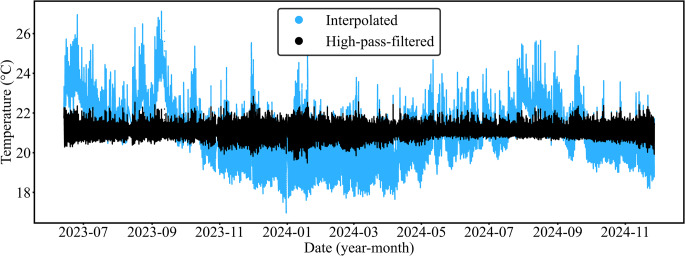
Temperature data from one home (blue), and resulting data after applying the high-pass filter (black). The data plot demonstrates the effect of removing frequencies lower than one cycle per day, and indicates a reduction in seasonal effects

Each time series was linearly interpolated to provide readings at 60s intervals, then processed using a high-pass filter to remove frequencies lower than one cycle per day. This frequency was chosen because:

- The characterisation of the sensor data was based on one day of data at a time, so lower frequencies would not be expected to be informative.
- The overall effect reduces seasonal differences (see Fig. 1).

Based on the pre-processed data, days were excluded from the analysis if there was an insufficient number of readings or readings were missing for three hours or more. Supplementary Materials Sect. 3 provides more detail.

For each day of sensor data, 25 characteristics (Table 2) were computed, as detailed below in Sect. 3.4.1 to 3.4.3

**Table 2:** Characteristics calculated for each day of sensor data

Section	Characteristic
3.4.1 Day descriptive statistics	day-mean
	standard deviation (SD)
	maximum value
	minimum value
	time of day of maximum
	time of day of minimum
	time of day absolute upper threshold first exceeded
	time of day absolute lower threshold first gone below
	time of day data-based upper threshold first exceeded
	time of day data-based lower threshold first gone below
3.4.2 Frequency and amplitudes	dominant frequency
	amplitude for 1 cycle per day
	amplitude for 1 cycle per hour
	amplitude mean
	amplitude SD
	amplitude minimum
	amplitude maximum
3.4.3 Deviation from modelled values	daily profile difference median
	daily profile difference minimum
	daily profile difference maximum
	daily profile difference 25th percentile
	daily profile difference 75th percentile
	time of day of the maximum daily profile difference
	daily profile RMSE
	LSTM RMSE

One day of sensor data spanned across 24 h and comprised 1440 readings (one per minute). The start time for a day was set to two hours earlier than the earliest time that the participant reported getting out of bed.

#### Day descriptive statistics

Characteristics were the mean average (day-mean), standard deviation (SD), maximum and minimum values, and times of day of the maximum and minimum. Times of day were also included for upper thresholds first exceeded, and lower thresholds first gone below. One set of thresholds was based on absolute values (Table 3), and a second set was data-based, defined as the mean ± 1 SD calculated over the complete time series for the participant.

**Table 3:** Absolute thresholds

	Upper threshold	Lower threshold
Electricity (W)	1500	200
CO_2_ (ppm)	600	401
Temperature (°C)	16	16
RH (%)	60	50

#### Frequency and amplitudes

A fast Fourier transform was applied to the sensor data for each day. Characteristics extracted were the most dominant frequency, amplitudes for the per-day and per-hour frequencies, and amplitude mean, SD, minimum and maximum.

The frequency representation was verified as an appropriate model for the data by reconstructing the data using an inverse Fourier transform. The characteristics were excluded from further analysis if the root mean square error (RMSE) between the original and reconstructed data was large (more than 1.96 SDs above the mean or 1.5 IQRs above the median of RMSEs for this participant).

#### Deviation from modelled values

The complete time series for each participant and sensor-measure was used to create two models of expected values for that home’s routine. The difference between the modelled (i.e., expected) values and the actual data provides a measure of how unusual that day is for that individual.

**Fig. 2:**
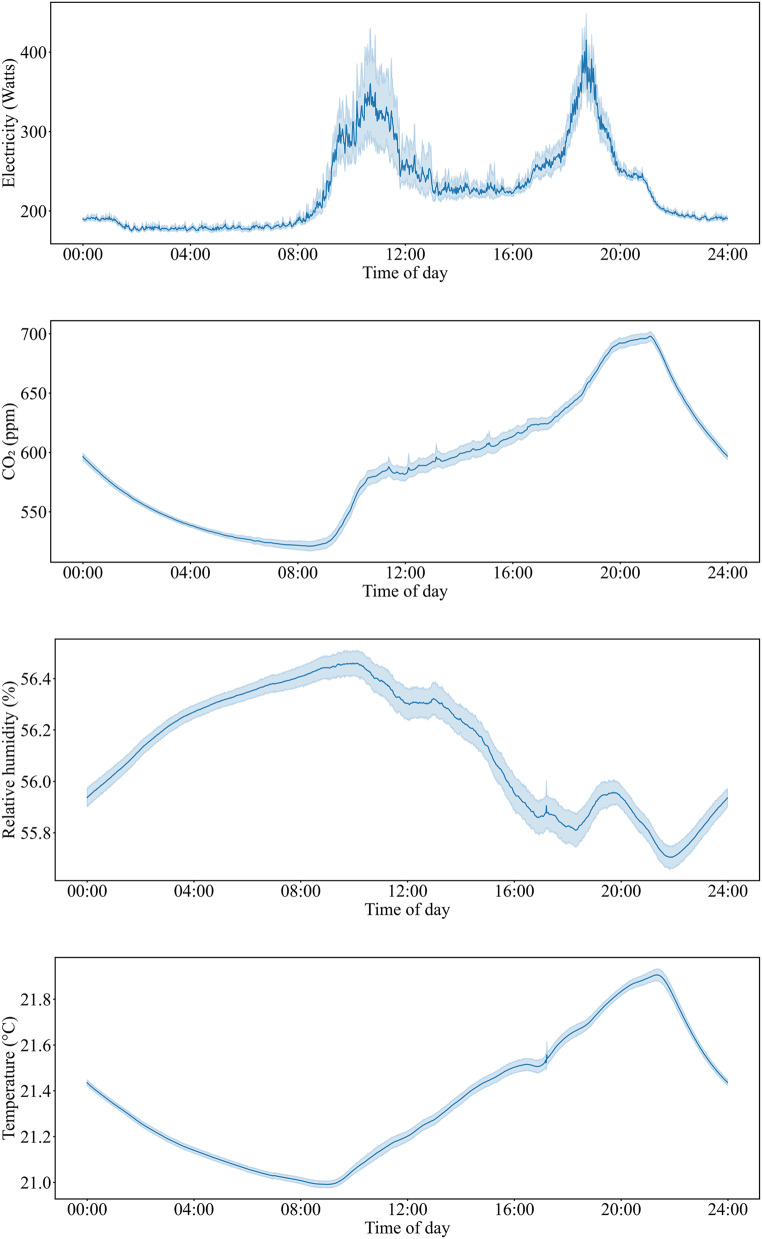
Daily profile of electrical power, CO_2_, relative humidity and temperature for one home. The different plots show how the levels for each sensor-measure change across the 24-hour period. Error bands represent the 95% confidence interval

The first model was a daily profile constructed by taking the mean average for each minute of the day over all days of sensor data. See Fig. 2. Differences between the sensor data for the day and the daily profile were calculated then scaled by the daily profile value, for each minute of the day. Characteristics calculated from the differences were mean, SD, median, minimum, maximum, 25th and 75th percentiles, the time of the maximum difference, and the RMSE between the sensor data and the daily profile. The mean and SD of differences were excluded from further analysis due to high correlation with the day-mean and the daily profile RMSE respectively (Pearson’s *r* > 0.97).

The second model utilised an LSTM with 24 timesteps on hourly means for the complete time series. The characteristic calculated was the RMSE between the original hourly data and the LSTM-modelled data for each day.

### Machine learning of occupant responses from sensor-data characteristics

**Fig. 3:**
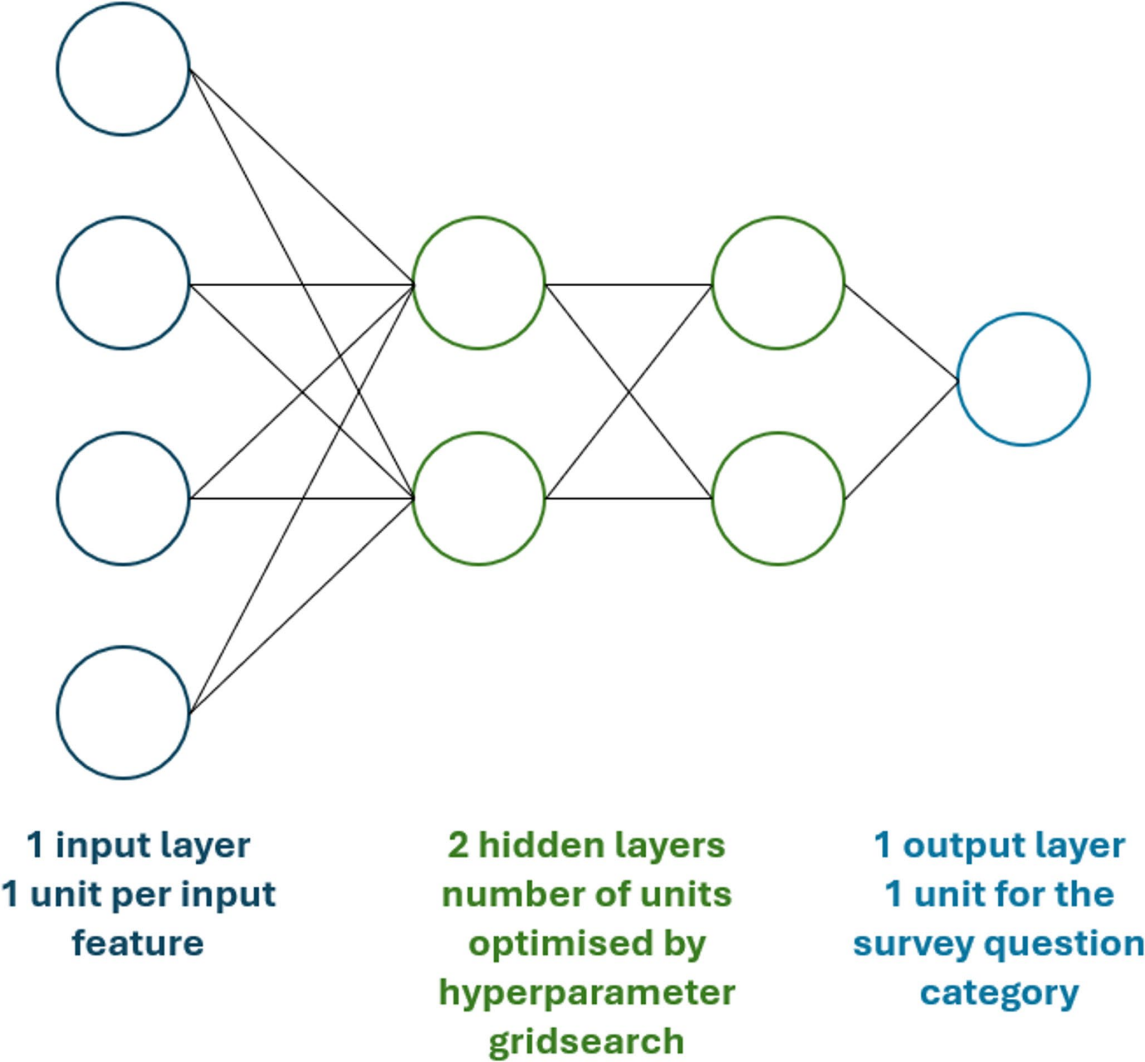
Representation of an example neural network, showing the input layer with one unit per input feature, two hidden layers, and the output layer with one unit

Multilayer perceptron regressor neural networks with four layers of units were implemented. Inputs were sensor-data characteristics and target outputs were occupant survey responses. See Fig. 3.

#### Input features

The potential input features to the network were the sensor-data characteristics for each of the four sensor-measures. Dummy variables were included for participant ID numbers and the days of the week.

Input features were removed if:

- They exhibited a low number of non-missing values (1 SD below the mean or more), because a missing feature value causes that sample to be excluded, and therefore impacts on the quantity of available data.
- They had a correlation with the ‘1’ response rates across the included participants, with a liberal rejection criterion (*p* ≤ 0.5). For example, if a participant with warm home frequently responds their health is worse than normal and a participant with cool home always responds their health is normal, then this negative correlation between mean temperature and ‘health worse’ response rate could cause learning based on a response bias, despite oversampling.

#### Output feature

Each network had a single output unit, with binary target output values from a given survey question category. Network outputs were continuous values and were thresholded to provide a binary response for comparison with the target output, as described in the Results (Sect.  4).

#### Hyperparameters

The number of hidden units in each of two hidden layers was chosen using a hyperparameter grid search for the number producing the best performance. Supplementary Materials Sect.  4 provides details.

#### Train and test

The dataset was split into 75% training samples and 25% test samples, stratified by health response (1 and 0) within each participant. Each type of network was implemented 20 times (hereafter called run). Synthetic minority oversampling technique (SMOTE) within each participant was applied to the training data to balance output classes (1 and 0). Each network was tested on data from participants whose data were included in the training data.

#### Network types

Separate networks were implemented for each survey question category using data from (1) all participants together, and (2) participants who responded to the survey at least 50 times, with a separate network for each participant.

#### Participant data exclusion criterion

All data from a participant were excluded if they had fewer than six responses of each class (1 and 0) in the training data. Different participants were therefore excluded depending on the survey question category, the subset of data selected for training, and the input features used.

#### Software tools

Data analyses were conducted using Python in Spyder, with NumPy, SciPy, Pandas, Scikit-learn, Imbalanced-learn, Tensorflow with Keras, Matplotlib and Seaborn [48–61].

## Results

To address the aim of testing whether changes in health and activities can be detected from sensor data, we assessed performance for neural networks to map from sensor-data characteristics to occupant responses about health and events.

Network outputs on a continuous scale were categorised as binary network responses for comparison with participant survey responses. The threshold for categorisation was optimised to provide the maximum *F1* score. Performance was compared against chance level, estimated by shuffling the network outputs for given inputs.

The main findings are summarised and interpreted in the next section. For detailed results, see Tables 4 and 5 for networks trained on data from all participants, Tables 6 and 7 for networks trained on data from the participant with most surveys answered, and Tables 8 and 9 for networks trained on a single participant’s data that gave the highest balanced accuracy above chance for the ‘health worse’ category. This participant answered the survey 50 times, and the daily profiles for their home are in Fig. 2. Supplementary Materials Sects.  5 and 6 provide additional details.

**Table 4:** Network performance on responses about health, trained on data from all included participants. Mean (minimum, maximum) and proportion above chance (**P**_**AC**_) over the 20 runs

Survey question category	Dataset Train or Test	BA Balanced accuracy on a scale of 0 to 1 = mean of true-positive and true-negative rates	AUC Area under the receiver operating characteristic curve	F1 score Overall accuracy of network ‘1’ responses	*N*_*p*_ Number of participants *N*_*r*_ Number of responses before oversampling *P*_r1_ Proportion of ‘1’ responses
Health worse	Train	0.87 (0.59, 0.93) ***P***_***AC***_ = 1.00	0.92 (0.65, 0.98) ***P***_***AC***_ = 1.00	0.88 (0.69, 0.93) ***P***_***AC***_ = 1.00	***N***_***p***_ = 13 ***N***_***r***_ = 1369 ***P***_***r1***_ = 0.30
	Test	0.54 (0.50, 0.58) ***P***_***AC***_ = 0.30	0.56 (0.48, 0.60) ***P***_***AC***_ = 0.70	0.48 (0.47, 0.50) ***P***_***AC***_ = 0.60	***N***_***p***_ = 13 ***N***_***r***_ = 472 ***P***_***r1***_ = 0.31
Health better	Train	0.84 (0.64, 0.97) ***P***_***AC***_ = 1.00	0.89 (0.70, 0.99) ***P***_***AC***_ = 1.00	0.86 (0.70, 0.97) ***P***_***AC***_ = 1.00	***N***_***p***_ = 9 ***N***_***r***_ = 1043 ***P***_***r1***_ = 0.20
	Test	0.58 (0.51, 0.63) ***P***_***AC***_ = 0.55	0.58 (0.50, 0.64) ***P***_***AC***_ = 0.55	0.37 (0.34, 0.40) ***P***_***AC***_ = 0.60	***N***_***p***_ = 9 ***N***_***r***_ = 356 ***P***_***r1***_ = 0.20
Existing health condition worse	Train	0.97 (0.66, 0.99) ***P***_***AC***_ = 1.00	0.99 (0.73, 1.00) ***P***_***AC***_ = 1.00	0.97 (0.72, 0.99) ***P***_***AC***_ = 1.00	***N***_***p***_ = 9 ***N***_***r***_ = 1049 ***P***_***r1***_ = 0.18
	Test	0.62 (0.57, 0.67) ***P***_***AC***_ = 0.95	0.63 (0.56, 0.70) ***P***_***AC***_ = 0.90	0.39 (0.35, 0.45) ***P***_***AC***_ = 0.95	***N***_***p***_ = 9 ***N***_***r***_ = 361 ***P***_***r1***_ = 0.19
Physical pain worse	Train	1.00 (0.99, 1.00) ***P***_***AC***_ = 1.00	1.00 (1.00, 1.00) ***P***_***AC***_ = 1.00	1.00 (0.99, 1.00) ***P***_***AC***_ = 1.00	***N***_***p***_ = 9 ***N***_***r***_ = 979 ***P***_***r1***_ = 0.21
	Test	0.58 (0.53, 0.67) ***P***_***AC***_ = 0.40	0.59 (0.51, 0.67) ***P***_***AC***_ = 0.85	0.39 (0.36, 0.47) ***P***_***AC***_ = 0.65	***N***_***p***_ = 9 ***N***_***r***_ = 334 ***P***_***r1***_ = 0.22
Mental health worse	Train	0.98 (0.88, 1.00) ***P***_***AC***_ = 1.00	0.99 (0.94, 1.00) ***P***_***AC***_ = 1.00	0.98 (0.89, 1.00) ***P***_***AC***_ = 1.00	***N***_***p***_ = 2 ***N***_***r***_ = 248 ***P***_***r1***_ = 0.20
	Test	0.75 (0.68, 0.83) ***P***_***AC***_ = 1.00	0.77 (0.65, 0.82) ***P***_***AC***_ = 0.95	0.60 (0.51, 0.73) ***P***_***AC***_ = 1.00	***N***_***p***_ = 2 ***N***_***r***_ = 84 ***P***_***r1***_ = 0.20

**Table 5:** Network performance on responses about events, trained on data from all included participants. Mean (minimum, maximum) and proportion above chance (**P**_**AC**_) over the 20 runs

Survey question category	Dataset Train or Test	BA Balanced accuracy on a scale of 0 to 1 = mean of true-positive and true-negative rates	AUC Area under the receiver operating characteristic curve	F1 score Overall accuracy of network ‘1’ responses	*N*_*p*_ Number of participants *N*_*r*_ Number of responses before oversampling *P*_r1_ Proportion of ‘1’ responses
Unusual event has happened	Train	0.91 (0.70, 0.98) ***P***_***AC***_ = 1.00	0.94 (0.77, 1.00) ***P***_***AC***_ = 1.00	0.92 (0.75, 0.98) ***P***_***AC***_ = 1.00	***N***_***p***_ = 5 ***N***_***r***_ = 614 ***P***_***r1***_ = 0.13
	Test	0.58 (0.50, 0.65) ***P***_***AC***_ = 0.10	0.56 (0.46, 0.68) ***P***_***AC***_ = 0.15	0.30 (0.26, 0.39) ***P***_***AC***_ = 0.20	***N***_***p***_ = 5 ***N***_***r***_ = 211 ***P***_***r1***_ = 0.15
Less time at home than usual	Train	0.79 (0.53, 1.00) ***P***_***AC***_ = 0.80	0.78 (0.51, 1.00) ***P***_***AC***_ = 0.80	0.82 (0.68, 1.00) ***P***_***AC***_ = 0.85	***N***_***p***_ = 1 ***N***_***r***_ = 25 ***P***_***r1***_ = 0.40
	Test	0.55 (0.50, 0.78) ***P***_***AC***_ = 0.05	0.37 (0.10, 0.80) ***P***_***AC***_ = 0.05	0.64 (0.62, 0.75) ***P***_***AC***_ = 0.10	***N***_***p***_ = 1 ***N***_***r***_ = 9 ***P***_***r1***_ = 0.44
More time at home than usual	Train	0.99 (0.98, 1.00) ***P***_***AC***_ = 1.00	1.00 (0.99, 1.00) ***P***_***AC***_ = 1.00	0.99 (0.98, 1.00) ***P***_***AC***_ = 1.00	***N***_***p***_ = 2 ***N***_***r***_ = 352 ***P***_***r1***_ = 0.05
	Test	0.62 (0.56, 0.69) ***P***_***AC***_ = 0.05	0.59 (0.49, 0.72) ***P***_***AC***_ = 0.15	0.23 (0.15, 0.43) ***P***_***AC***_ = 0.15	***N***_***p***_ = 2 ***N***_***r***_ = 120 ***P***_***r1***_ = 0.07
Maintenance or building work	Train	0.96 (0.94, 0.98) ***P***_***AC***_ = 1.00	0.98 (0.95, 1.00) ***P***_***AC***_ = 1.00	0.96 (0.94, 0.98) ***P***_***AC***_ = 1.00	***N***_***p***_ = 1 ***N***_***r***_ = 125 ***P***_***r1***_ = 0.10
	Test	0.74 (0.59, 0.87) ***P***_***AC***_ = 0.50	0.72 (0.53, 0.85) ***P***_***AC***_ = 0.50	0.47 (0.29, 0.67) ***P***_***AC***_ = 0.65	***N***_***p***_ = 1 ***N***_***r***_ = 43 ***P***_***r1***_ = 0.12
A visitor to the home	Train	0.99 (0.91, 1.00) ***P***_***AC***_ = 1.00	0.99 (0.95, 1.00) ***P***_***AC***_ = 1.00	0.99 (0.91, 1.00) ***P***_***AC***_ = 1.00	***N***_***p***_ = 2 ***N***_***r***_ = 295 ***P***_***r1***_ = 0.08
	Test	0.66 (0.59, 0.81) ***P***_***AC***_ = 0.20	0.67 (0.54, 0.78) ***P***_***AC***_ = 0.50	0.32 (0.22, 0.44) ***P***_***AC***_ = 0.50	***N***_***p***_ = 2 ***N***_***r***_ = 100 ***P***_***r1***_ = 0.09

**Table 6:** Network performance on responses about health, trained on data from the participant with most surveys answered. Mean (minimum, maximum) and proportion above chance (**P**_**AC**_) over the 20 runs

Survey question category	Dataset Train or Test	BA Balanced accuracy on a scale of 0 to 1 = mean of true-positive and true-negative rates	AUC Area under the receiver operating characteristic curve	F1 score Overall accuracy of network ‘1’ responses	*N*_*p*_ Number of participants *N*_*r*_ Number of responses before oversampling *P*_r1_ Proportion of ‘1’ responses
Health worse	Train	1.00 (0.96, 1.00) **P**_**AC**_ = 1.00	1.00 (0.98, 1.00) **P**_**AC**_ = 1.00	1.00 (0.96, 1.00) **P**_**AC**_ = 1.00	***N***_***p***_ = 1 ***N***_***r***_ = 103 ***P***_***r1***_ = 0.20
	Test	0.61 (0.50, 0.82) **P**_**AC**_ = 0.05	0.52 (0.22, 0.72) **P**_**AC**_ = 0.05	0.44 (0.36, 0.71) **P**_**AC**_ = 0.05	***N***_***p***_ = 1 ***N***_***r***_ = 36 ***P***_***r1***_ = 0.22
Health better	Train	1.00 (0.99, 1.00) **P**_**AC**_ = 1.00	1.00 (0.99, 1.00) **P**_**AC**_ = 1.00	1.00 (0.99, 1.00) **P**_**AC**_ = 1.00	***N***_***p***_ = 1 ***N***_***r***_ = 99 ***P***_***r1***_ = 0.12
	Test	0.84 (0.68, 0.93) **P**_**AC**_ = 0.80	0.86 (0.69, 0.94) **P**_**AC**_ = 0.85	0.62 (0.47, 0.75) **P**_**AC**_ = 0.85	***N***_***p***_ = 1 ***N***_***r***_ = 35 ***P***_***r1***_ = 0.14
Existing health condition worse	Train	0.99 (0.95, 1.00) **P**_**AC**_ = 1.00	1.00 (0.97, 1.00) **P**_**AC**_ = 1.00	0.99 (0.95, 1.00) **P**_**AC**_ = 1.00	***N***_***p***_ = 1 ***N***_***r***_ = 102 Pr1 = 0.18
	Test	0.59 (0.50, 0.69) **P**_**AC**_ = 0.00	0.46 (0.31, 0.64) **P**_**AC**_ = 0.00	0.34 (0.29, 0.43) **P**_**AC**_ = 0.00	***N***_***p***_ = 1 ***N***_***r***_ = 35 ***P***_***r1***_ = 0.17
Physical pain worse	Train	1.00 (1.00, 1.00) **P**_**AC**_ = 1.00	1.00 (1.00, 1.00) **P**_**AC**_ = 1.00	1.00 (1.00, 1.00) **P**_**AC**_ = 1.00	***N***_***p***_ = 1 ***N***_***r***_ = 92 ***P***_***r1***_ = 0.07
	Test	0.66 (0.57, 0.90) **P**_**AC**_ = 0.05	0.51 (0.29, 0.84) **P**_**AC**_ = 0.05	0.29 (0.20, 0.50) **P**_**AC**_ = 0.10	***N***_***p***_ = 1 ***N***_***r***_ = 32 ***P***_***r1***_ = 0.09

**Table 7:** Network performance on responses about events, trained on data from the participant with most surveys answered. Mean (minimum, maximum) and proportion above chance (**P**_**AC**_) over the 20 runs

Survey question category	Dataset Train or Test	BA Balanced accuracy on a scale of 0 to 1 = mean of true-positive and true-negative rates	AUC Area under the receiver operating characteristic curve	F1 score Overall accuracy of network ‘1’ responses	*N*_*p*_ Number of participants *N*_*r*_ Number of responses before oversampling *P*_r1_ Proportion of ‘1’ responses
Unusual event has happened	Train	1.00 (0.98, 1.00) **P**_**AC**_ = 1.00	1.00 (0.99, 1.00) **P**_**AC**_ = 1.00	1.00 (0.98, 1.00) **P**_**AC**_ = 1.00	***N***_***p***_ = 1 ***N***_***r***_ = 110 ***P***_***r1***_ = 0.10
	Test	0.74 (0.59, 0.94) **P**_**AC**_ = 0.35	0.71 (0.50, 0.93) **P**_**AC**_ = 0.35	0.47 (0.29, 0.67) **P**_**AC**_ = 0.40	***N***_***p***_ = 1 ***N***_***r***_ = 37 ***P***_***r1***_ = 0.11
Less time at home than usual	Train	1.00 (1.00, 1.00) **P**_**AC**_ = 1.00	1.00 (1.00, 1.00) **P**_**AC**_ = 1.00	1.00 (1.00, 1.00) **P**_**AC**_ = 1.00	***N***_***p***_ = 1 ***N***_***r***_ = 107 ***P***_***r1***_ = 0.06
	Test	0.82 (0.69, 0.97) **P**_**AC**_ = 0.25	0.85 (0.44, 0.97) **P**_**AC**_ = 0.30	0.54 (0.16, 0.67) **P**_**AC**_ = 0.55	***N***_***p***_ = 1 ***N***_***r***_ = 36 ***P***_***r1***_ = 0.06

**Table 8:** Network performance on responses about health, trained on data from the participant with the highest balanced accuracy above chance for the ‘health worse’ category. Mean (minimum, maximum) and proportion above chance (**P**_**AC**_) over the 20 runs

Survey question category	Dataset Train or Test	BA Balanced accuracy on a scale of 0 to 1 = mean of true-positive and true-negative rates	AUC Area under the receiver operating characteristic curve	F1 score Overall accuracy of network ‘1’ responses	*N*_*p*_ Number of participants *N*_*r*_ Number of responses before oversampling *P*_r1_ Proportion of ‘1’ responses
Health worse	Train	0.98 (0.93, 1.00) **P**_**AC**_ = 1.00	1.00 (0.97, 1.00) **P**_**AC**_ = 1.00	0.98 (0.93, 1.00) **P**_**AC**_ = 1.00	***N***_***p***_ = 1 ***N***_***r***_ = 96 ***P***_***r1***_ = 0.26
	Test	0.75 (0.58, 0.88) **P**_**AC**_ = 0.60	0.76 (0.54, 0.93) **P**_**AC**_ = 0.70	0.62 (0.47, 0.82) **P**_**AC**_ = 0.65	***N***_***p***_ = 1 ***N***_***r***_ = 33 ***P***_***r1***_ = 0.27
Existing health condition worse	Train	1.00 (0.99, 1.00) **P**_**AC**_ = 1.00	1.00 (1.00, 1.00) **P**_**AC**_ = 1.00	1.00 (0.99, 1.00) **P**_**AC**_ = 1.00	***N***_***p***_ = 1 ***N***_***r***_ = 82 ***P***_***r1***_ = 0.11
	Test	0.80 (0.67, 0.98) **P**_**AC**_ = 0.35	0.78 (0.55, 0.97) **P**_**AC**_ = 0.50	0.52 (0.38, 0.89) **P**_**AC**_ = 0.40	***N***_***p***_ = 1 ***N***_***r***_ = 29 ***P***_***r1***_ = 0.14
Physical pain worse	Train	1.00 (0.99, 1.00) **P**_**AC**_ = 1.00	1.00 (1.00, 1.00) **P**_**AC**_ = 1.00	1.00 (0.99, 1.00) **P**_**AC**_ = 1.00	***N***_***p***_ = 1 ***N***_***r***_ = 88 ***P***_***r1***_ = 0.17
	Test	0.84 (0.71, 0.98) **P**_**AC**_ = 0.80	0.85 (0.73, 0.99) **P**_**AC**_ = 0.80	0.74 (0.55, 0.92) **P**_**AC**_ = 1.00	***N***_***p***_ = 1 ***N***_***r***_ = 31 ***P***_***r1***_ = 0.19
Mental health worse	Train	1.00 (0.99, 1.00) **P**_**AC**_ = 1.00	1.00 (0.99, 1.00) **P**_**AC**_ = 1.00	1.00 (0.99, 1.00) **P**_**AC**_ = 1.00	***N***_***p***_ = 1 ***N***_***r***_ = 89 ***P***_***r1***_ = 0.20
	Test	0.87 (0.75, 1.00) **P**_**AC**_ = 0.90	0.91 (0.73, 1.00) **P**_**AC**_ = 0.95	0.78 (0.50, 1.00) **P**_**AC**_ = 0.95	***N***_***p***_ = 1 ***N***_***r***_ = 30 ***P***_***r1***_ = 0.20

**Table 9:** Network performance on responses about events, trained on data from the participant with the highest balanced accuracy above chance for the ‘health worse’ category. Mean (minimum, maximum) and proportion above chance (**P**_**AC**_) over the 20 runs

Survey question category	Dataset Train or Test	BA Balanced accuracy on a scale of 0 to 1 = mean of true-positive and true-negative rates	AUC Area under the receiver operating characteristic curve	F1 score Overall accuracy of network ‘1’ responses	*N*_*p*_ Number of participants *N*_*r*_ Number of responses before oversampling *P*_r1_ Proportion of ‘1’ responses
Unusual event has happened	Train	1.00 (0.99, 1.00) **P**_**AC**_ = 1.00	1.00 (0.99, 1.00) **P**_**AC**_ = 1.00	1.00 (0.99, 1.00) **P**_**AC**_ = 1.00	***N***_***p***_ = 1 ***N***_***r***_ = 96 ***P***_***r1***_ = 0.16
	Test	0.72 (0.54, 0.88) **P**_**AC**_ = 0.25	0.69 (0.41, 0.87) **P**_**AC**_ = 0.30	0.51 (0.32, 0.67) **P**_**AC**_ = 0.45	***N***_***p***_ = 1 ***N***_***r***_ = 34 ***P***_***r1***_ = 0.18
A visitor to the home	Train	1.00 (0.98, 1.00) **P**_**AC**_ = 1.00	1.00 (1.00, 1.00) **P**_**AC**_ = 1.00	1.00 (0.98, 1.00) **P**_**AC**_ = 1.00	***N***_***p***_ = 1 ***N***_***r***_ = 93 ***P***_***r1***_ = 0.10
	Test	0.76 (0.62, 0.93) **P**_**AC**_ = 0.40	0.73 (0.57, 0.95) **P**_**AC**_ = 0.40	0.49 (0.32, 0.75) **P**_**AC**_ = 0.50	***N***_***p***_ = 1 ***N***_***r***_ = 32 ***P***_***r1***_ = 0.12

## Discussion

Addressing our main aim, we found evidence that indoor environment and electricity data can capture changes in routine that are associated with changes in health or events in the home. These sensors could therefore be used in future to support healthy independent living.

### Interpretation of findings

Here, we summarise and interpret the main findings. We report the balanced accuracy (*BA*) on the test data, unseen during training, and the proportion of runs with *BA* above chance (*P*_*AC*_).

Detecting that an existing health condition was worse than normal (*BA* = 62%, *P*_*AC*_ = 0.95) gave higher performance than detecting general worse health (*BA* = 54%, *P*_*AC*_= 0.30). Responses for an existing condition form a subset of the responses for worse health more generally. When health is worse generally, behaviours may vary depending on the effects of the worse health. However, behaviours in response to an existing health condition are likely to be more consistent over time, and therefore provide consistent changes in sensor-data characteristics.

Detection of worse mental health gave the highest performance (*BA* = 75%, *P*_*AC*_ = 1.00), while performance was low for physical pain (*BA* = 58%, *P*_*AC*_ = 0.40). For mental health, only two participants provided sufficient numbers of responses, so behaviour in response to their health is likely to be more consistent throughout the dataset.

For unusual events, only five participants provided sufficient numbers of responses. For specific events, two participants provided sufficient responses for a visitor to the home and for more time at home, and only one participant for maintenance and for less time at home. It appears that participants were more likely to respond about their health than events, perhaps because health was the focus of this study. For networks trained with more than one participant, performance was highest for detecting a visitor to the home (*BA* = 66%, *P*_*AC*_ = 0.20).

Networks trained using an individual participant’s data typically exhibited higher performance than networks using data from all participants together. This is perhaps not surprising given that preferences, routines and behaviours will vary between individuals. In addition, sensors were not always in the same location across homes. Participants of the qualitative focus groups and interviews noted individual differences in their own daily routines, such as batch cooking or not using the kettle if going out for breakfast. Modelling of sensor data at the individual level allows for variations in the participant’s routine to be taken into account, because the sensor-data characteristics will also vary, so absolute values that appear unusually low or high are not necessarily unusual relative to the distribution of values for that individual.

The individual network that gave the best performance above chance for worse health (*BA* = 75%, *P*_*AC*_ = 0.60) was for the participant that gave the highest proportion of ‘worse’ responses (0.26). The network therefore had more information for training and for subsequent generalisation to the test data. Networks using this participant’s data also gave the highest performance for worse existing health condition (*BA* = 80%, *P*_*AC*_ = 0.35, except for one participant with a low ‘worse’ response rate of 0.06), mental health (*BA* = 87%, *P*_*AC*_ = 0.90) and physical pain (*BA* = 84%, *P*_*AC*_ = 0.80).

### Strengths and Novelty

The main strength of this feasibility study was the use of off-the-shelf sensors that are already used for building, comfort, or energy management in many homes in the UK and beyond. However, we have shown that these existing data streams can also be used to detect changes in health. Furthermore, these sensors are easy to install, relatively inexpensive, and meet a need for non-intrusive monitoring (e.g., [40, 62]).

To our knowledge this study recruited more participants and for a longer period of time than any other previous comparable study. Unlike previous studies, the detection of worsening health was not targeted to a particular condition, and the methods we tested for detection were therefore not tailored to monitoring specific behaviours. The reasoning was to test whether information can be inferred using a broad-brush approach, and we have shown that there is indeed evidence of health changes in sensor data, which provides a strong foundation for future research and application.

### Limitations

The main limitation was few responses from most participants. Even when participants answered the survey often, the usefulness of the data was limited due to a lack of variation in responses.

In particular, for networks trained on data from an individual data, the number of samples was low compared to the number of parameters in the network. This imbalance can result in overfitting, which results in high performance on training data, but poor generalisation to test data [63]. Indeed, training performance is high in the current study, suggesting overfitting, but performance on test data is still good, suggesting that overfitting is not a significant shortcoming. Other work has also found that the relationship between numbers of samples and network parameters need not be as stringent as generally suggested in order to provide good generalisation [64].

Generalisation of findings are limited to occupants who live alone. They may also be limited to occupants with comparable housing and health, which should be a consideration in future work.

### Implications and Future work

Beyond detecting changes in health, the combination of relative humidity, electrical power, temperature, and carbon dioxide can be used to infer a person’s daily routine. Supplementary Materials Sect.  7 provides an example. Used alongside contextual information, notifications about changes could be tailored to specific health conditions, specific activities of interest, or service provider needs. With more data collected for specific health conditions or about a person’s typical routine, cluster analysis could be employed to provide groups of individuals, with each group assigned a template model for use when the system is first installed. In future, it may also be possible to predict worsening health earlier and proactively support independent living at home.

Work with stakeholders suggests that these findings, together with future research on earlier prediction of poor health, will be informative to local councils for providing directed homecare and healthy housing, organisations that support older adults’ independent living, and health and care providers for scheduling home visits, tailoring personalised care, or prevention of health issues (e.g., [7]).

## Conclusion

Findings from this feasibility study provide evidence that data from environmental and electrical-power sensors can be used to detect changes in occupant health. These sensors, which are readily available and already installed in many UK homes, could therefore be used in the future to support independent living at home. When indicators of worsening health are detected, interventions could be made, including external support from service providers or recommendations for behavioural change, to help avoid the issue worsening or slow down progression. Such systems have the potential to address the ever-increasing pressure on health and care provision and effectiveness.

## Supplementary

Below is the link to the electronic supplementary material.

Supplementary Material 1 (PDF 738 KB)
