# Supplementary material for: Detecting changes in health and daily activities using environmental and electricity sensors and machine learning
Source: Health Technol (Berl). 2026 May 26;16(4):757–70. doi: 10.1007/s12553-026-01082-x (PMC13303745; doi:10.1007/s12553-026-01082-x)
Supplement: Supplementary file 1 — Supplementary Material 1 (PDF 738 KB) [file 12553_2026_1082_MOESM1_ESM.pdf]

Supplementary materials: Detecting changes in health and daily activities using environmental and electricity sensors and machine learning

Journal name: *Health and Technology*

Authors: Tamaryn Menneer[12], Tim Walker[1], Karen Spooner[13], Emma Bland[1], Lucia Pratto[1], Ian Wellaway[4], Mark England[3], Richard A. Sharpe[15], Catherine Leyshon[6], Markus Mueller[27].

[1]European Centre for Environment and Human Health, University of Exeter Medical School, University of Exeter, Penryn Campus, UK.
[2]Environment and Sustainability Institute, University of Exeter, Penryn Campus, UK.
[3]Coastline Housing Ltd, Barncoose Gateway Park, Redruth, UK.
[4]Research IT, University of Exeter, Streatham Campus, UK.
[5]Wellbeing & Public Health, Cornwall Council, County Hall, Truro, Cornwall, UK.
[6]Centre for Geography and Environmental Science, University of Exeter, Penryn Campus, UK.
[7]Department of Earth and Environmental Science, Faculty of Environment, Science and Economy, University of Exeter, Penryn Campus, UK.

Corresponding author: Tamaryn Menneer, t.s.i.menneer@exeter.ac.uk

1 Health and activities data collection

1.1 Optional survey questions

The optional survey was sent to participants approximately once a week, via text message or email, with a link to a Microsoft Form containing the questions in Table S1 and Table S2. Three further questions were included to allow for other reasons that unusual patterns might be seen in the environment or electricity use, but responses were not used in the current study.

Responses for the questions about the previous week were back-propagated to the correct date relating to the response, so that those responses were paired with sensor-data characteristics for the appropriate day. If responses were conflicting, then ‘worse’ ‘better’ ‘yes’ and ‘maybe’ responses were prioritised over ‘normal’ or ‘no’, with the reasoning being that someone would be less likely to falsely remember a difference than to forget that it occurred. For example, a participant responds ‘normal health’ for the previous week, but the response actually provided on one of those dates was ‘worse’.

Table S1: Optional survey questions and response options about health

	**Question**	**Response options**
Q1	How is your health today? (Compared with what is approximately normal for you.)	○ Much worse than normal<br>○ Slightly worse than normal<br>○ Normal for me<br>○ Slightly better than normal<br>○ Much better than normal<br>○ Prefer not to answer
Q2<br>If ‘worse’ response for Q1	Is there a reason that your health is worse than normal today? You can select multiple options.	○ Physical pain<br>○ Mental health<br>○ An existing health condition has been worse than normal<br>○ Prefer not to answer<br>○ Other:
Q3	In the past week, not including today, how has your health been? (Compared with what is approximately normal for you.)	As for Q1
Q4<br>If ‘worse’ response for Q3		As for Q2
Q5<br>If ‘worse’ response for Q3	Do you know the day your health was worse than normal? You can select more than one day.	○ Sunday<br>○ Monday<br>○ Tuesday<br>○ Wednesday<br>○ Thursday<br>○ Friday<br>○ Saturday<br>○ Prefer not to answer

Table S2: Optional survey questions and response options about unusual events

	**Question**	**Response options**
Q6	Is there an unusual event in your home today?	○ Yes<br>○ No<br>○ Maybe<br>○ Prefer not to answer
Q7<br>If 'Yes' or 'Maybe' response to Q6	What is the unusual event today? You can select multiple answers.	○ A visitor to my home<br>○ Maintenance or building work<br>○ More time spent at home than usual<br>○ Less time spent at home than usual<br>○ Prefer not to answer<br>○ Other:
Q8	In the past week, not including today, has there been an unusual event in your home?	As for Q6
Q9	In the past week, what was the unusual event? You can select multiple answers.	As for Q7
Q10	In the past week, do you know the day of the unusual event? You can select more than one day.	○ Sunday<br>○ Monday<br>○ Tuesday<br>○ Wednesday<br>○ Thursday<br>○ Friday<br>○ Saturday<br>○ Prefer not to answer

1.2 Health and daily routine questionnaire questions

In the questionnaire taken at beginning, middle and end of the participant's involvement in the project, participants were asked "What time do you typically get out of bed?". If a range was provided, then the earliest time was chosen. For three participants, there was no definitive response (e.g., "depends as I am so unwell", "varies due to health"), so a default start time of 04:00 was used, chosen as sufficiently early to start monitoring for activity in the home, but late enough that it would be unlikely to capture activity from the preceding night. The start time for the sensor data for the day was set to two hours earlier than the earliest reported time, so that monitoring started for the day before the participant got up from bed.

2 Sensor technical specifications

The temperature and relative humidity (RH) sensor was battery-powered. The carbon dioxide (CO_2) sensor and gateway were mains-powered. The electricity sensor comprised a current-transformer clip (CT-clip) and a sensor to transmit the readings to the gateway. Two types of electricity sensor were used across different homes, due to availability of each type during the recruitment phase. One type of electricity sensor required an additional mains-powered receiver attached to the gateway, which was used in 22 of the 36 homes.

Readings from the sensors were transmitted to a gateway via Ultra High Frequency radio waves, and transmitted to the sensor providers' data storage via any available Global System for Mobile Communications networks using a Subscriber Identity Module (i.e., SIM) card. The SIM card avoided reliance on home internet connection. Table S3 provides equipment specifications.

Table S3: Sensor equipment specifications

**Equipment**	**Interval between readings (seconds)**	**Typical accuracy**	**Type of operation**	**Approximate dimensions (mm)**	**Manufacturer and model**
Gateway	30	Not applicable	Readings received via Ultra High Frequency radio (433MHz); GSM-enabled (Global System for Mobile Communications)	75 × 65 x 25	Purrmetrix PMTX020064
Temperature and relative humidity (RH) sensor	30-60	Temperature: ± 0.5%<br><br>RH: ±3%	Temperature: Band-gap<br><br>RH: Capacitive	60 × 22 × 8	Purrmetrix PMTX010003
Carbon dioxide (CO_2) sensor	30-60	± (50ppm + 3% reading value)	Nondispersive infrared	90 × 48 × 20	Purrmetrix PMTX010008
Electrical current sensor A	30-60	± 2.5%	Current transformer clamp	90 × 24 x 10	Purrmetrix PMTX01012801
Electrical current sensor B	12-60	± 5%	Current transformer clamp; Separate receiver, connected to the GSM-enabled gateway with ethernet cable	Sensor: 95 × 65 x 28<br><br>Receiver: 110 × 40 × 22	2 Save Energy Ltd OWL Intuition-e

3 Sensor data cleaning

The first two hours of temperature and RH readings after sensor installation were removed from the time series. For CO_2, the first two days were removed. These time periods followed sensor provider guidance to ensure correct calibration in the new environment.

Each time series typically contained readings at a higher frequency than the specification of the sensor frequency. Readings were resampled to provide an interval of 60s, and missing values were linearly interpolated to provide one value per minute, before applying the high-pass filter.

Days were excluded from the analysis if (1) the number of readings for the day was half the number of minimum readings expected or less, where the number of expected readings is 1440 (24 hours × 60 minutes); or (2) the time series for the day contained a gap in readings of three hours or more in duration.

4 Network hyperparameters

Default scikit-learn settings for the weight optimisation solver (Adam optimiser) and L2 regularisation term strength were used (0.0001) for the regressor neural network.

A halving grid search was used to optimise the number of hidden units in each of two hidden layers The search was conducted using the complete dataset, with the synthetic minority oversampling technique (SMOTE) applied within each participant to balance output classes. Grid search was conducted 10 times, and the best performing hyperparameters were used to train the network.

The maximum number of hidden units was the mean of the number of input and output units, and the minimum was one third of the maximum. The grid also contained three evenly-spaced intervening values, giving a grid size of 5×5=25 for two hidden layers.

5 Performance metrics

We calculated the true positive rate (*TPR*) and the true negative rate (*TNR*), which are combined to give the accuracy balanced across the number of 1 and 0 survey responses (*BA*). We also report *AUC* and *F1*, which provide a measure of accuracy of 1 responses by combining true and false positives. Table S4 and Table S5 present the formulae and more detail.

Performance values were compared with chance-level values. Theoretically, chance-level balanced accuracy (*BA*) should be 0.5, given that the *TPR* and *TNR* are weighted by the number of 1 and 0 observed responses. However, for datasets with low numbers of responses, a change in response (from response of 1 to response of 0 for example) can disproportionately influence the overall *BA*. The same influences apply to *AUC*. Chance-level *F1* is also not 0.5, because the numbers of 1 and 0 observed responses differ. Chance-level performance metrics were estimated by calculating the measures for the shuffled set of network outputs, and selecting the 1/0 threshold that maximised each metric. This process was conducted 10 times and the mean values for *BA*, *AUC* and *F1* were taken as the chance levels. The standard deviation of the 10 values was used to measure how far the performance metric value for the network model response fell from chance-level value. It is worth noting that the chance-level *BA* is based on a 1/0 threshold to maximise *BA*, while the *BA* reported for the network model is based on the 1/0 threshold that maximised *F1*. The same threshold was used for both measures in order to provide consistent 1/0 responses from the network model across all performance measures.

Table S4: Different types of responses and the corresponding accuracy rates.

<table>
<tr><td></td><td></td><td colspan="2">Observed survey response</td><td></td></tr>
<tr><td></td><td></td><td>1</td><td>0</td><td></td></tr>
<tr><td rowspan="2">Network response</td><td>1</td><td>True positive (TP)</td><td>False positive (FP)</td><td>Precision = $\frac{\text{Number of } TP\text{s}}{\text{Number of 1 network responses}}$</td></tr>
<tr><td>0</td><td>False negative (FN)</td><td>True negative (TN)</td><td></td></tr>
<tr><td></td><td></td><td>TP rate (TPR) = $\frac{\text{Number of } TP\text{s}}{\text{Number of 1 survey responses}}$</td><td>TN rate (TNR) = $\frac{\text{Number of } TN\text{s}}{\text{Number of 0 survey responses}}$</td><td></td></tr>
</table>

Table S5: Performance measures to assess the accuracy of the network.

**Performance measure**	**Represents**
Balanced accuracy (*BA*) = $\frac{TPR+TNR}{2}$	Overall accuracy of the network responses balanced across the number of 1 and 0 survey responses.
Area under receiver operating characteristic (*ROC*) curve = *AUC*	The *ROC* curve represents a trade-off between the *TPR* and the *FP* rate (*FPR*) as a function of the threshold for binary output classes. The *AUC* represents gains in the *TPR* relative to the *FPR*, with larger values representing larger gains.
F1 = $2\left(\frac{Precision*TPR}{Precision+TPR}\right)$	A combination of the true positive rate and the precision, thereby accounting for observed 1 responses that are missed as well as correctly identified 1s. Overall, *F1* provides measure of accuracy of 1 responses from the network model.

6 Results tables

Table S6 to Table S11provide performance information in addition to the metrics reported in the main paper.

***BA*, *AUC* and *F1* as reported in the main paper.** *BA*, *AUC*, *F1* and P_{AC} values are repeated, as reported in the main paper, which are calculated from network responses using a 1/0 threshold that was optimised for the corresponding dataset (train or test set).

***BA* and *F1* for test data using the training threshold.** *BA*, *F1* and P_{AC} are reported for the test set using the 1/0 threshold optimised on the training set. The optimised training threshold was higher than the optimised test threshold for all network types, leading to lower true positive rates for test data when using the training threshold, and *F1* values often lower than chance level. The *AUC* is independent of a threshold for binary network responses.

Additional performance metrics. These tables also include the overall accuracy (*OA*), not balanced across response type (1 or 0), the true positive rate (*TPR*) and true negative rate (*TNR*).

*Table S6: Network performance on responses about health, trained on data from all included participants. Mean (minimum, maximum) and proportion above chance (**P_{AC}**) over the 20 runs.*

**Survey question category**	**Dataset**<br><br>Train or Test	***BA***<br><br>Balanced accuracy<br>= mean of true-positive and true-negative rates	***AUC***<br><br>Area under the receiver operating characteristic curve	***F1*** score<br><br>Overall accuracy of network '1' responses	***N_p*** Number of participants<br><br>***N_r*** Number of responses before oversampling<br><br>***P_{r1}*** Proportion of '1' responses	***OA***<br><br>Overall accuracy, not balanced across response type	***TPR***<br><br>True positive rate	***TNR***<br><br>True negative rate
**Health worse**	Train	0.87<br>(0.59, 0.93)<br>P_{AC} = 1.00	0.92<br>(0.65, 0.98)<br>P_{AC} = 1.00	0.88<br>(0.69, 0.93)<br>P_{AC} = 1.00	N_p = 13<br>N_r = 1369<br>P_{r1} = 0.30	0.87<br>(0.59, 0.93)	0.91<br>(0.87, 0.94)	0.82<br>(0.29, 0.93)
	Test	0.54<br>(0.50, 0.58)<br>P_{AC} = 0.30	0.56<br>(0.48, 0.60)<br>P_{AC} = 0.70	0.48<br>(0.47, 0.50)<br>P_{AC} = 0.60	N_p = 13<br>N_r = 472<br>P_{r1} = 0.31	0.39<br>(0.31, 0.51)	0.92<br>(0.73, 1.00)	0.15<br>(0.00, 0.42)
	Test with train threshold	0.54<br>(0.47, 0.58)<br>P_{AC} = 0.45	-	0.40<br>(0.33, 0.44)<br>P_{AC} = 0.00	-	0.56<br>(0.37, 0.63)	0.48<br>(0.36, 0.74)	0.60<br>(0.21, 0.72)
**Health better**	Train	0.84<br>(0.64, 0.97)<br>P_{AC} = 1.00	0.89<br>(0.70, 0.99)<br>P_{AC} = 1.00	0.86<br>(0.70, 0.97)<br>P_{AC} = 1.00	N_p = 9<br>N_r = 1043<br>P_{r1} = 0.20	0.84<br>(0.64, 0.97)	0.91<br>(0.85, 0.97)	0.76<br>(0.43, 0.98)
	Test	0.58<br>(0.51, 0.63)<br>P_{AC} = 0.55	0.58<br>(0.50, 0.64)<br>P_{AC} = 0.55	0.37<br>(0.34, 0.40)<br>P_{AC} = 0.60	N_p = 9<br>N_r = 356<br>P_{r1} = 0.20	0.45<br>(0.23, 0.64)	0.79<br>(0.53, 1.00)	0.37<br>(0.04, 0.67)
	Test with train threshold	0.56<br>(0.49, 0.60)<br>P_{AC} = 0.25	-	0.31<br>(0.21, 0.38)<br>P_{AC} = 0.20	-	0.61<br>(0.42, 0.73)	0.46<br>(0.21, 0.81)	0.65<br>(0.37, 0.84)

Table S6: Continued.

	Dataset	*BA*	*AUC*	*F1* score	N_p N_r P_{r1}	*OA*	*TPR*	*TNR*
**Existing health condition worse**	Train	0.97 (0.66, 0.99) P_{AC} = 1.00	0.99 (0.73, 1.00) P_{AC} = 1.00	0.97 (0.72, 0.99) P_{AC} = 1.00	N_p = 9 N_r = 1049 P_{r1} = 0.18	0.97 (0.66, 0.99)	0.98 (0.87, 1.00)	0.96 (0.46, 0.99)
	Test	0.62 (0.57, 0.67) P_{AC} = 0.95	0.63 (0.56, 0.70) P_{AC} = 0.90	0.39 (0.35, 0.45) P_{AC} = 0.95	N_p = 9 N_r = 361 P_{r1} = 0.19	0.61 (0.37, 0.78)	0.64 (0.47, 0.93)	0.60 (0.24, 0.85)
	Test with train threshold	0.59 (0.55, 0.64) P_{AC} = 0.50	-	0.34 (0.30, 0.41) P_{AC} = 0.30	-	0.71 (0.51, 0.75)	0.39 (0.30, 0.66)	0.78 (0.48, 0.84)
**Physical pain worse**	Train	1.00 (0.99, 1.00) P_{AC} = 1.00	1.00 (1.00, 1.00) P_{AC} = 1.00	1.00 (0.99, 1.00) P_{AC} = 1.00	N_p = 9 N_r = 979 P_{r1} = 0.21	1.00 (0.99, 1.00)	1.00 (0.99, 1.00)	1.00 (0.99, 1.00)
	Test	0.58 (0.53, 0.67) P_{AC} = 0.40	0.59 (0.51, 0.67) P_{AC} = 0.85	0.39 (0.36, 0.47) P_{AC} = 0.65	N_p = 9 N_r = 334 P_{r1} = 0.22	0.46 (0.28, 0.75)	0.79 (0.50, 0.99)	0.36 (0.08, 0.82)
	Test with train threshold	0.56 (0.52, 0.63) P_{AC} = 0.30	-	0.33 (0.23, 0.43) P_{AC} = 0.10	-	0.67 (0.62, 0.75)	0.38 (0.19, 0.49)	0.75 (0.66, 0.86)
**Mental health worse**	Train	0.98 (0.88, 1.00) P_{AC} = 1.00	0.99 (0.94, 1.00) P_{AC} = 1.00	0.98 (0.89, 1.00) P_{AC} = 1.00	N_p = 2 N_r = 248 P_{r1} = 0.20	0.98 (0.88, 1.00)	0.99 (0.94, 1.00)	0.97 (0.83, 1.00)
	Test	0.75 (0.68, 0.83) P_{AC} = 1.00	0.77 (0.65, 0.82) P_{AC} = 0.95	0.60 (0.51, 0.73) P_{AC} = 1.00	N_p = 2 N_r = 84 P_{r1} = 0.20	0.83 (0.74, 0.90)	0.62 (0.41, 0.88)	0.88 (0.72, 0.97)
	Test with train threshold	0.70 (0.59, 0.77) P_{AC} = 0.80	-	0.52 (0.32, 0.67) P_{AC} = 0.95	-	0.81 (0.74, 0.88)	0.52 (0.24, 0.82)	0.88 (0.72, 0.99)

*Table S7: Network performance on responses about events, trained on data from all included participants. Mean (minimum, maximum) and proportion above chance (**P_{AC}**) over the 20 runs.*

	Dataset	*BA*	*AUC*	*F1* score	N_p N_r P_{r1}	*OA*	*TPR*	*TNR*
**Unusual event has happened**	Train	0.91 (0.70, 0.98) P_{AC} = 1.00	0.94 (0.77, 1.00) P_{AC} = 1.00	0.92 (0.75, 0.98) P_{AC} = 1.00	N_p = 5 N_r = 614 P_{r1} = 0.13	0.91 (0.70, 0.98)	0.94 (0.81, 0.98)	0.88 (0.53, 0.98)
	Test	0.58 (0.50, 0.65) P_{AC} = 0.10	0.56 (0.46, 0.68) P_{AC} = 0.15	0.30 (0.26, 0.39) P_{AC} = 0.20	N_p = 5 N_r = 211 P_{r1} = 0.15	0.54 (0.15, 0.81)	0.65 (0.35, 1.00)	0.52 (0.01, 0.88)
	Test with train threshold	0.54 (0.44, 0.64) P_{AC} = 0.05	-	0.22 (0.03, 0.37) P_{AC} = 0.05	-	0.70 (0.50, 0.79)	0.31 (0.03, 0.68)	0.77 (0.47, 0.89)
**Less time at home than usual**	Train	0.79 (0.53, 1.00) P_{AC} = 0.80	0.78 (0.51, 1.00) P_{AC} = 0.80	0.82 (0.68, 1.00) P_{AC} = 0.85	N_p = 1 N_r = 25 P_{r1} = 0.40	0.79 (0.53, 1.00)	0.93 (0.73, 1.00)	0.64 (0.07, 1.00)
	Test	0.55 (0.50, 0.78) P_{AC} = 0.05	0.37 (0.10, 0.80) P_{AC} = 0.05	0.64 (0.62, 0.75) P_{AC} = 0.10	N_p = 1 N_r = 9 P_{r1} = 0.44	0.50 (0.44, 0.78)	0.97 (0.75, 1.00)	0.12 (0.00, 0.80)
	Test with train threshold	0.40 (0.20, 0.68) P_{AC} = 0.00	-	0.33 (0.00, 0.67) P_{AC} = 0.00	-	0.40 (0.22, 0.67)	0.36 (0.00, 0.75)	0.43 (0.20, 0.80)
**More time at home than usual**	Train	0.99 (0.98, 1.00) P_{AC} = 1.00	1.00 (0.99, 1.00) P_{AC} = 1.00	0.99 (0.98, 1.00) P_{AC} = 1.00	N_p = 2 N_r = 352 P_{r1} = 0.05	0.99 (0.98, 1.00)	1.00 (0.99, 1.00)	0.99 (0.97, 1.00)
	Test	0.62 (0.56, 0.69) P_{AC} = 0.05	0.59 (0.49, 0.72) P_{AC} = 0.15	0.23 (0.15, 0.43) P_{AC} = 0.15	N_p = 2 N_r = 120 P_{r1} = 0.07	0.78 (0.42, 0.94)	0.44 (0.12, 0.88)	0.80 (0.38, 1.00)
	Test with train threshold	0.52 (0.46, 0.61) P_{AC} = 0.00	-	0.09 (0.00, 0.31) P_{AC} = 0.10	-	0.89 (0.85, 0.94)	0.09 (0.00, 0.25)	0.95 (0.90, 1.00)

Table S7: Continued.

	**Dataset**	***BA***	***AUC***	***F1* score**	***N_p N_r P_{r1}***	***OA***	***TPR***	***TNR***
**Maintenance or building work**	Train	0.96 (0.94, 0.98) P_{AC} = 1.00	0.98 (0.95, 1.00) P_{AC} = 1.00	0.96 (0.94, 0.98) P_{AC} = 1.00	N_p = 1 N_r = 125 P_{r1} = 0.10	0.96 (0.94, 0.98)	0.99 (0.96, 1.00)	0.93 (0.89, 0.96)
	Test	0.74 (0.59, 0.87) P_{AC} = 0.50	0.72 (0.53, 0.85) P_{AC} = 0.50	0.47 (0.29, 0.67) P_{AC} = 0.65	N_p = 1 N_r = 43 P_{r1} = 0.12	0.82 (0.53, 0.93)	0.65 (0.20, 1.00)	0.84 (0.50, 1.00)
	Test with train threshold	0.64 (0.43, 0.82) P_{AC} = 0.20	-	0.32 (0.00, 0.53) P_{AC} = 0.25	-	0.81 (0.72, 0.91)	0.41 (0.00, 0.80)	0.86 (0.74, 0.97)
**A visitor to the home**	Train	0.99 (0.91, 1.00) P_{AC} = 1.00	0.99 (0.95, 1.00) P_{AC} = 1.00	0.99 (0.91, 1.00) P_{AC} = 1.00	N_p = 2 N_r = 295 P_{r1} = 0.08	0.99 (0.91, 1.00)	0.99 (0.96, 1.00)	0.98 (0.86, 1.00)
	Test	0.66 (0.59, 0.81) P_{AC} = 0.20	0.67 (0.54, 0.78) P_{AC} = 0.50	0.32 (0.22, 0.44) P_{AC} = 0.50	N_p = 2 N_r = 100 P_{r1} = 0.09	0.79 (0.59, 0.92)	0.52 (0.22, 0.89)	0.81 (0.57, 0.98)
	Test with train threshold	0.55 (0.40, 0.64) P_{AC} = 0.00	-	0.18 (0.00, 0.38) P_{AC} = 0.10	-	0.84 (0.73, 0.91)	0.18 (0.00, 0.33)	0.91 (0.80, 0.98)

*Table S8: Network performance on responses about health, trained on data from the participant with most surveys answered. Mean (minimum, maximum) and proportion above chance (**P_{AC}**) over the 20 runs.*

**Survey question category**	**Dataset**<br><br>Train or Test	***BA***<br><br>Balanced accuracy = mean of true-positive and true-negative rates	***AUC***<br><br>Area under the receiver operating characteristic curve	***F1*** score<br><br>Overall accuracy of network '1' responses	N_p Number of participants<br><br>N_r Number of responses before oversampling<br><br>P_{r1} Proportion of '1' responses	***OA***<br><br>Overall accuracy, not balanced across response type	***TPR***<br><br>True positive rate	***TNR***<br><br>True negative rate
**Health worse**	Train	1.00 (0.96, 1.00) P_{AC} = 1.00	1.00 (0.98, 1.00) P_{AC} = 1.00	1.00 (0.96, 1.00) P_{AC} = 1.00	N_p = 1 N_r = 103 P_{r1} = 0.20	1.00 (0.96, 1.00)	1.00 (0.98, 1.00)	0.99 (0.95, 1.00)
	Test	0.61 (0.50, 0.82) P_{AC} = 0.05	0.52 (0.22, 0.72) P_{AC} = 0.05	0.44 (0.36, 0.71) P_{AC} = 0.05	N_p = 1 N_r = 36 P_{r1} = 0.22	0.51 (0.22, 0.86)	0.78 (0.25, 1.00)	0.44 (0.00, 1.00)
	Test with train threshold	0.52 (0.38, 0.63) P_{AC} = 0.00	-	0.21 (0.00, 0.43) P_{AC} = 0.00	-	0.69 (0.47, 0.81)	0.21 (0.00, 0.50)	0.83 (0.46, 0.96)
**Health better**	Train	1.00 (0.99, 1.00) P_{AC} = 1.00	1.00 (0.99, 1.00) P_{AC} = 1.00	1.00 (0.99, 1.00) P_{AC} = 1.00	N_p = 1 N_r = 99 P_{r1} = 0.12	1.00 (0.99, 1.00)	1.00 (0.99, 1.00)	0.99 (0.98, 1.00)
	Test	0.84 (0.68, 0.93) P_{AC} = 0.80	0.86 (0.69, 0.94) P_{AC} = 0.85	0.62 (0.47, 0.75) P_{AC} = 0.85	N_p = 1 N_r = 35 P_{r1} = 0.14	0.86 (0.71, 0.94)	0.83 (0.40, 1.00)	0.86 (0.67, 1.00)
	Test with train threshold	0.63 (0.48, 0.77) P_{AC} = 0.10	-	0.35 (0.00, 0.60) P_{AC} = 0.35	-	0.86 (0.80, 0.91)	0.32 (0.00, 0.60)	0.95 (0.87, 1.00)

Table S8: Continued.

	**Dataset**	***BA***	***AUC***	***F1*** score	***N_p N_r P_{r1}***	***OA***	***TPR***	***TNR***
**Existing health condition worse**	Train	0.99 (0.95, 1.00) P_{AC} = 1.00	1.00 (0.97, 1.00) P_{AC} = 1.00	0.99 (0.95, 1.00) P_{AC} = 1.00	N_p = 1 N_r = 102 Pr1= 0.18	0.99 (0.95, 1.00)	1.00 (0.99, 1.00)	0.99 (0.90, 1.00)
	Test	0.59 (0.50, 0.69) P_{AC} = 0.00	0.46 (0.31, 0.64) P_{AC} = 0.00	0.34 (0.29, 0.43) P_{AC} = 0.00	N_p = 1 N_r = 35 P_{r1} = 0.17	0.44 (0.17, 0.80)	0.82 (0.33, 1.00)	0.36 (0.00, 0.90)
	Test with train threshold	0.49 (0.38, 0.65) P_{AC} = 0.00	-	0.12 (0.00, 0.40) P_{AC} = 0.00	-	0.71 (0.51, 0.80)	0.15 (0.00, 0.50)	0.83 (0.59, 0.97)
**Physical pain worse**	Train	1.00 (1.00, 1.00) P_{AC} = 1.00	1.00 (1.00, 1.00) P_{AC} = 1.00	1.00 (1.00, 1.00) P_{AC} = 1.00	N_p = 1 N_r = 92 P_{r1} = 0.07	1.00 (1.00, 1.00)	1.00 (1.00, 1.00)	1.00 (1.00, 1.00)
	Test	0.66 (0.57, 0.90) P_{AC} = 0.05	0.51 (0.29, 0.84) P_{AC} = 0.05	0.29 (0.20, 0.50) P_{AC} = 0.10	N_p = 1 N_r = 32 P_{r1} = 0.09	0.63 (0.28, 0.94)	0.70 (0.33, 1.00)	0.62 (0.21, 1.00)
	Test with train threshold	0.50 (0.48, 0.50) P_{AC} = 0.00	-	0.00 (0.00, 0.00) P_{AC} = 0.00	-	0.88 (0.88, 0.88)	0.00 (0.00, 0.00)	0.97 (0.97, 0.97)

*Table S9: Network performance on responses about events, trained on data from the participant with most surveys answered. Mean (minimum, maximum) and proportion above chance (**P_{AC}**) over the 20 runs.*

	**Dataset**	***BA***	***AUC***	***F1*** score	***N_p N_r P_{r1}***	***OA***	***TPR***	***TNR***
**Unusual event has happened**	Train	1.00 (0.98, 1.00) P_{AC} = 1.00	1.00 (0.99, 1.00) P_{AC} = 1.00	1.00 (0.98, 1.00) P_{AC} = 1.00	N_p = 1 N_r = 110 P_{r1} = 0.10	1.00 (0.98, 1.00)	1.00 (1.00, 1.00)	0.99 (0.96, 1.00)
	Test	0.74 (0.59, 0.94) P_{AC} = 0.35	0.71 (0.50, 0.93) P_{AC} = 0.35	0.47 (0.29, 0.67) P_{AC} = 0.40	N_p = 1 N_r = 37 P_{r1} = 0.11	0.84 (0.68, 0.95)	0.62 (0.25, 1.00)	0.86 (0.67, 1.00)
	Test with train threshold	0.55 (0.45, 0.72) P_{AC} = 0.00	-	0.17 (0.00, 0.50) P_{AC} = 0.10	-	0.87 (0.81, 0.92)	0.18 (0.00, 0.50)	0.95 (0.91, 1.00)
**Less time at home than usual**	Train	1.00 (1.00, 1.00) P_{AC} = 1.00	1.00 (1.00, 1.00) P_{AC} = 1.00	1.00 (1.00, 1.00) P_{AC} = 1.00	N_p = 1 N_r = 107 P_{r1} = 0.06	1.00 (1.00, 1.00)	1.00 (1.00, 1.00)	1.00 (0.99, 1.00)
	Test	0.82 (0.69, 0.97) P_{AC} = 0.25	0.85 (0.44, 0.97) P_{AC} = 0.30	0.54 (0.16, 0.67) P_{AC} = 0.55	N_p = 1 N_r = 36 P_{r1} = 0.06	0.89 (0.42, 0.97)	0.75 (0.50, 1.00)	0.90 (0.38, 1.00)
	Test with train threshold	0.57 (0.49, 0.75) P_{AC} = 0.00	-	0.19 (0.00, 0.67) P_{AC} = 0.25	-	0.96 (0.92, 0.97)	0.43 (0.00, 0.50)	0.99 (0.97, 1.00)

*Table S10: Network performance on responses about health, trained on data from the participant with the highest balanced accuracy above chance for the 'health worse' category. Mean (minimum, maximum) and proportion above chance (**P_{AC}**) over the 20 runs.*

**Survey question category**	**Dataset**<br><br>Train or Test	***BA***<br><br>Balanced accuracy = mean of true-positive and true-negative rates	***AUC***<br><br>Area under the receiver operating characteristic curve	***F1*** score<br><br>Overall accuracy of network '1' responses	***N_p*** Number of participants<br><br>***N_r*** Number of responses before oversampling<br><br>***P_{r1}*** Proportion of '1' responses	***OA***<br><br>Overall accuracy, not balanced across response type	***TPR***<br><br>True positive rate	***TNR***<br><br>True negative rate
**Health worse**	Train	0.98 (0.93, 1.00) **P_{AC}** = 1.00	1.00 (0.97, 1.00) **P_{AC}** = 1.00	0.98 (0.93, 1.00) **P_{AC}** = 1.00	***N_p*** = 1 ***N_r*** = 96 ***P_{r1}*** = 0.26	0.98 (0.93, 1.00)	0.99 (0.94, 1.00)	0.97 (0.87, 1.00)
	Test	0.75 (0.58, 0.88) **P_{AC}** = 0.60	0.76 (0.54, 0.93) **P_{AC}** = 0.70	0.62 (0.47, 0.82) **P_{AC}** = 0.65	***N_p*** = 1 ***N_r*** = 33 ***P_{r1}*** = 0.27	0.72 (0.39, 0.91)	0.82 (0.44, 1.00)	0.68 (0.17, 0.96)
	Test with train threshold	0.67 (0.56, 0.81) **P_{AC}** = 0.30	-	0.51 (0.31, 0.70) **P_{AC}** = 0.35	-	0.74 (0.64, 0.85)	0.51 (0.22, 0.89)	0.82 (0.67, 1.00)
**Existing health condition worse**	Train	1.00 (0.99, 1.00) **P_{AC}** = 1.00	1.00 (1.00, 1.00) **P_{AC}** = 1.00	1.00 (0.99, 1.00) **P_{AC}** = 1.00	***N_p*** = 1 ***N_r*** = 82 ***P_{r1}*** = 0.11	1.00 (0.99, 1.00)	1.00 (1.00, 1.00)	1.00 (0.99, 1.00)
	Test	0.80 (0.67, 0.98) **P_{AC}** = 0.35	0.78 (0.55, 0.97) **P_{AC}** = 0.50	0.52 (0.38, 0.89) **P_{AC}** = 0.40	***N_p*** = 1 ***N_r*** = 29 ***P_{r1}*** = 0.14	0.78 (0.55, 0.97)	0.82 (0.50, 1.00)	0.77 (0.48, 0.96)
	Test with train threshold	0.53 (0.46, 0.73) **P_{AC}** = 0.00	-	0.12 (0.00, 0.57) **P_{AC}** = 0.05	-	0.83 (0.79, 0.90)	0.13 (0.00, 0.50)	0.94 (0.88, 0.96)

Table S10: Continued.

	Dataset	*BA*	*AUC*	*F1* score	N_p N_r P_{r1}	*OA*	*TPR*	*TNR*
**Physical pain worse**	Train	1.00 (0.99, 1.00) P_{AC} = 1.00	1.00 (1.00, 1.00) P_{AC} = 1.00	1.00 (0.99, 1.00) P_{AC} = 1.00	N_p = 1 N_r = 88 P_{r1} = 0.17	1.00 (0.99, 1.00)	1.00 (1.00, 1.00)	1.00 (0.97, 1.00)
	Test	0.84 (0.71, 0.98) P_{AC} = 0.80	0.85 (0.73, 0.99) P_{AC} = 0.80	0.74 (0.55, 0.92) P_{AC} = 1.00	N_p = 1 N_r = 31 P_{r1} = 0.19	0.90 (0.81, 0.97)	0.75 (0.50, 1.00)	0.94 (0.84, 1.00)
	Test with train threshold	0.78 (0.58, 0.98) P_{AC} = 0.55	-	0.64 (0.29, 0.92) P_{AC} = 0.75	-	0.87 (0.77, 0.97)	0.62 (0.17, 1.00)	0.94 (0.80, 1.00)
**Mental health worse**	Train	1.00 (0.99, 1.00) P_{AC} = 1.00	1.00 (0.99, 1.00) P_{AC} = 1.00	1.00 (0.99, 1.00) P_{AC} = 1.00	N_p = 1 N_r = 89 P_{r1} = 0.20	1.00 (0.99, 1.00)	1.00 (1.00, 1.00)	1.00 (0.99, 1.00)
	Test	0.87 (0.75, 1.00) P_{AC} = 0.90	0.91 (0.73, 1.00) P_{AC} = 0.95	0.78 (0.50, 1.00) P_{AC} = 0.95	N_p = 1 N_r = 30 P_{r1} = 0.20	0.90 (0.60, 1.00)	0.83 (0.67, 1.00)	0.91 (0.50, 1.00)
	Test with train threshold	0.80 (0.50, 0.96) P_{AC} = 0.75	-	0.69 (0.00, 0.91) P_{AC} = 0.80	-	0.90 (0.80, 0.97)	0.67 (0.33, 1.00)	0.96 (0.88, 1.00)

*Table S11: Network performance on responses about events, trained on data from the participant with the highest balanced accuracy above chance for the 'health worse' category. Mean (minimum, maximum) and proportion above chance (**P_{AC}**) over the 20 runs.*

	Dataset	*BA*	*AUC*	*F1* score	N_p N_r P_{r1}	*OA*	*TPR*	*TNR*
**Unusual event has happened**	Train	1.00 (0.99, 1.00) P_{AC} = 1.00	1.00 (0.99, 1.00) P_{AC} = 1.00	1.00 (0.99, 1.00) P_{AC} = 1.00	N_p = 1 N_r = 96 P_{r1} = 0.16	1.00 (0.99, 1.00)	1.00 (1.00, 1.00)	1.00 (0.98, 1.00)
**Unusual event has happened**	Test	0.72 (0.54, 0.88) P_{AC} = 0.25	0.69 (0.41, 0.87) P_{AC} = 0.30	0.51 (0.32, 0.67) P_{AC} = 0.45	N_p = 1 N_r = 34 P_{r1} = 0.18	0.74 (0.24, 0.91)	0.68 (0.33, 1.00)	0.76 (0.07, 1.00)
**Unusual event has happened**	Test with train threshold	0.60 (0.46, 0.75) P_{AC} = 0.10	-	0.33 (0.00, 0.67) P_{AC} = 0.20	-	0.80 (0.71, 0.91)	0.30 (0.00, 0.67)	0.91 (0.79, 1.00)
**A visitor to the home**	Train	1.00 (0.98, 1.00) P_{AC} = 1.00	1.00 (1.00, 1.00) P_{AC} = 1.00	1.00 (0.98, 1.00) P_{AC} = 1.00	N_p = 1 N_r = 93 P_{r1} = 0.10	1.00 (0.98, 1.00)	1.00 (1.00, 1.00)	1.00 (0.96, 1.00)
**A visitor to the home**	Test	0.76 (0.62, 0.93) P_{AC} = 0.40	0.73 (0.57, 0.95) P_{AC} = 0.40	0.49 (0.32, 0.75) P_{AC} = 0.50	N_p = 1 N_r = 32 P_{r1} = 0.12	0.78 (0.47, 0.94)	0.74 (0.25, 1.00)	0.78 (0.39, 1.00)
**A visitor to the home**	Test with train threshold	0.57 (0.45, 0.75) P_{AC} = 0.00	-	0.23 (0.00, 0.67) P_{AC} = 0.10	-	0.88 (0.78, 0.94)	0.22 (0.00, 0.50)	0.97 (0.89, 1.00)

7 Daily profile example

Figure S1 shows the daily profiles for one home for each sensor-measure, together with a single day on which the sensor-data characteristics were unusual.

From the daily profiles (in blue), the following daily routine can be inferred for this occupant, by using the combination of relative humidity, electrical power, temperature, and carbon dioxide, together with some contextual information about the individual. Every day, Mr. Jones gets up at about 7.30am, makes himself a cup of tea, then has a shower. Just after midday, Mr. Jones has lunch, and then he spends a few hours in the living room, watching the television, and perhaps listening to the radio. At around 4pm, Mr. Jones makes himself a snack and some tea, then he has an afternoon nap. At around 8pm, Mr. Jones cooks his evening meal, and at 10pm he turns up the heating before he goes to bed at about 11.30pm.

The plots for the single day (in red) show the typical morning peaks in relative humidity and carbon dioxide have shifted to later in the day, while the electricity and temperate data do not significantly differ from the normal pattern. These patterns suggest that Mr. Jones had his shower later in the day than normal.

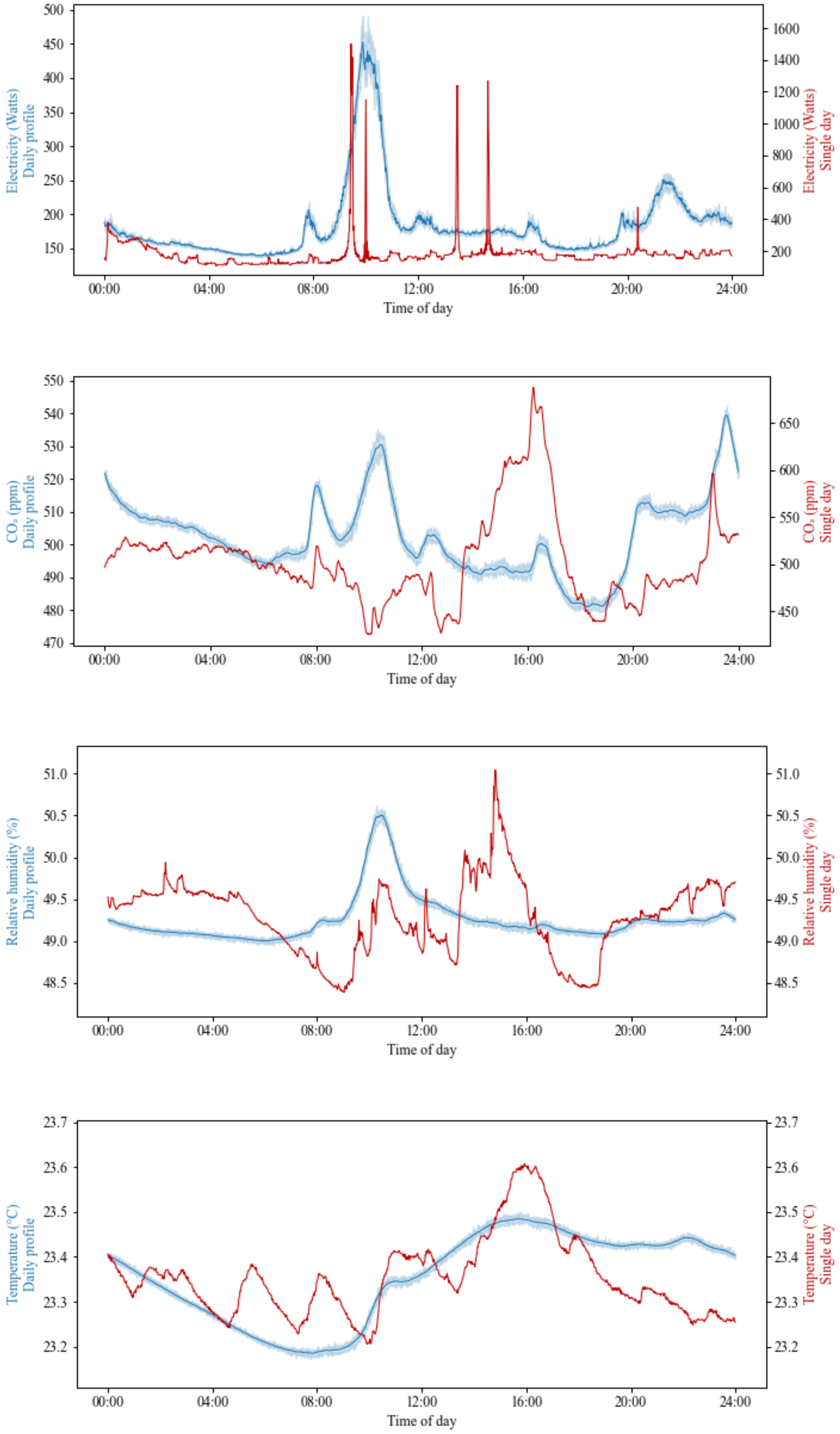

Figure S1: Daily profiles in blue of electrical power, CO_2, relative humidity and temperature for one home, plotted on the left-hand axis. Error bands represent the 95% confidence interval. A single day of data is presented in red for each sensor-measure, plotted on the right-hand vertical axis.
